# Effects of breast structure on high-intensity focused ultrasound focal error

**DOI:** 10.1186/s40349-018-0111-9

**Published:** 2018-06-20

**Authors:** Kohei Okita, Ryuta Narumi, Takashi Azuma, Hidemi Furusawa, Junichi Shidooka, Shu Takagi, Yoichiro Matsumoto

**Affiliations:** 1 Department of Mechanical Engineering, College of Industrial Technology, Nihon University, 1-2-1 Izumi-cho, Narashino, Chiba, 275-8575 Japan; 2 Department of Bioengineering, School of Engineering, The University of Tokyo, 7-3-1 Hongo, Bunkyo-ku, Tokyo, 113-8654 Japan; 3 Breastopia Namba Hospital, 2-112-1 Maruyama, Miyazaki-shi, Miyazaki, 880-0052 Japan; 4 Department of Mechanical Engineering, School of Engineering, The University of Tokyo, 7-3-1 Hongo, Bunkyo-ku, Tokyo, 113-8654 Japan; 5 Kawaguchi Kogyo General Hospital, 1-18-25 Aoki, Kawaguchi, Saitama, 332-0031 Japan; 6 Tokyo University of Science, 1-3 Kagurazaka, Shinjuku-ku, Tokyo, 162-8601 Japan

**Keywords:** High-intensity focused ultrasound, Breast cancer, Numerical simulation

## Abstract

**Background:**

The development of imaging technologies and breast cancer screening allowed early detection of breast cancers. High-intensity focused ultrasound (HIFU) is a non-invasive cancer treatment, but the success of HIFU ablation was depending on the system type, imaging technique, ablation protocol, and patient selection. Therefore, we aimed to determine the relationship between breast tissue structure and focal error during breast cancer HIFU treatment.

**Methods:**

Numerical simulations of the breast cancer HIFU ablation were performed using digital breast phantoms constructed using the magnetic resonance imaging data obtained from 12 patients.

**Results:**

The focal shapes were distorted despite breast tissue representing soft tissue. Focal errors are caused by the complex distribution of fibroglandular tissue, and they depend on the target position and the arrangement of the transducer. We demonstrated that the focusing ratio increases with the decrease in the local acoustic inhomogeneity, implying that it may be used as an indicator to reduce the HIFU focal error depending on the breast structure.

**Conclusions:**

The obtained results demonstrated that the focal error observed during the breast cancer HIFU treatment is highly dependent on the structure of fibroglandular tissue. The optimal arrangement of the transducer to the target can be obtained by minimizing the local acoustic inhomogeneity before the breast cancer HIFU treatment.

## Background

Breast cancer incidence is increasing worldwide, and this disease represents the cancer type with the highest morbidity rates in women, accounting for over 40% morbidity among women over 40 years of age in Japan. The standard care for localized breast cancer includes breast-conserving therapy, which is an invasive treatment [1]. Therefore, less or non-invasive treatments, such as cryoablation, radiofrequency ablation, laser ablation, microwave ablation, and high-intensity focused ultrasound (HIFU) ablation, have been developed with the aim of treating early-stage breast cancer patients. HIFU is a non-invasive thermal ablation treatment. Ultrasound emitted from a transducer outside the body propagates through tissues, focusing on the target tissue. The focused acoustic energy is converted into thermal energy, elevating the temperature inside the target tissue to the tumor necrosis-inducing temperature within a few seconds, without damaging adjacent tissues [2].

The development of imaging technologies and breast cancer screening have allowed the earlier and more sensitive detection of breast cancers [3]. Ultrasound computed tomography (USCT) was developed as an early-stage diagnostic technique for breast cancer [4, 5]. For tumors that are too small to determine whether they are benign [6] or malignant, HIFU may represent an effective treatment because it allows the ablation of a small regions and is a non-invasive and repeatable treatment.

HIFU has been used for breast cancer treatment [7–12], and its rate of successful ablation was reported to range from 20 to 100%, depending on the system type, imaging technique, ablation protocol, and patient selection. The size of tumor, distance to the skin and pectoral muscle, and position inside the breast should be determined prior to the treatment to assess whether a patient is eligible for the HIFU treatment. Numerical simulation can be a useful tool to determine these aspects.

Current HIFU devices mainly employ magnetic resonance imaging (MRI) for the guidance of treatment. MRI enables not only pre- and post-contrast imaging of cancer tissue for the validation of the treatment but also real-time temperature monitoring during the treatment. Although the correlation between the applied energy and tumor necrosis size during the HIFU treatment was shown to be good, no correlation was found between the applied energy and the increase in the temperature [13]. This suggests the presence of the focal error, which is caused by the ultrasound wave reflection and refraction due to the acoustic inhomogeneity of a body. The improvement of ultrasound wave focusing should therefore allow a more efficient and safer treatment. Furthermore, cooling times and breathing correction contribute the most to the treatment costs [14]. MR-HIFU ablation is currently not a cost-effective alternative to the breast-conserving therapy, and ultrasound imaging guidance is expected to lower the cost of HIFU treatments.

Since it is not possible to observe the ultrasound wave propagation, numerical simulation represents an effective tool for the understanding of the applied ultrasound field and for the development of therapeutic ultrasound devices as well as ultrasound imaging techniques. Numerical simulation of the HIFU treatment was applied and the focus control was examined using a phased-array transducer [15, 16]. The shape of the superficial tissue layers was shown to play a significant role in determining the shape of the focal spot. It was reported that increasing the complexity of the model did not help achieve a better agreement between the simulation and measurements, which highlights the importance of validating acoustical simulations with experimental data [17]. Numerical simulation can accurately reproduce the ultrasound propagation through inhomogeneous media with a known shape and physical properties; however, the modeling and numerical simulation of the HIFU treatment are still under development due to the complexity and unknown physical properties of the tissues.

In this study, the breast models constructed using the MRI data obtained from 12 patients were employed for the breast cancer HIFU treatment simulations. The relationship between the focal error during the breast cancer HIFU treatment and the breast structure was examined numerically. Initially, we performed breast modeling, followed by the application of the HIFU simulator to reproduce the ultrasound propagation through the tissue.

## Methods

### Breast modeling

A digital phantom of breast tissues was generated from the MRI data, which was assumed to comprise three tissue types, including skin, fat, and fibroglandular tissue because a focus was assigned away from pectoralis muscle or lung. The procedure applied for the generation of a breast phantom is as follows:

1. Three-dimensional volume data of the brightness value were obtained by stacking the MRI data, which were three-dimensionally denoised through a median filter (3 × 3 × 3).
2. Segmentation of breast and air was performed. Initially, a histogram of brightness was produced from the volume data, followed by the selection of the minimum value between the first and second local maximum values to obtain the threshold brightness value and distinguish between breast and air. Finally, the brightness values in the volume data lower than the threshold brightness value were set to 0, representing the regions of air.
3. We identified the interface between breast tissue and air regions. Considering an average brightness value of the breast tissue region, in which the brightness values were larger than 0, 50% of the average brightness value was selected as the threshold value. The interface between the breast tissue and the air region was detected using the line scan from the air to the breast region based on the selected threshold value, and the brightness value of the interface was temporarily set to − 1.
4. Skin region (thickness, 1.5 mm) was added from the identified interface to the air region in the normal direction of the interface. The brightness of the skin in the volume data was temporarily set to − 1. Therefore, the brightness volume data were segmented into the air region, skin region, and internal breast tissue by setting the brightness values to 0, − 1, and the original brightness values, respectively.

These intermediate volume data were employed as the input data for the breast cancer HIFU treatment simulation, where the air region was treated as the water region. Therefore, the HIFU simulator can distinguish fat from fibroglandular tissue using the selected threshold brightness values. The threshold brightness value used to distinguish fat and fibroglandular tissues was selected as a fat ratio from the histogram of the brightness values inside breast tissue except for pectoralis muscle and lung. Afterward, the digital breast phantom was generated and water, skin, fat, and fibroglandular tissues were segmented. Benign or malignant breast tissues was segmented as fibroglandular tissue even though depending on the brightness threshold. The physical properties of media shown in Table 1 were employed for the simulation of ultrasound propagation.

**Table 1:** Physical properties of media

	Skin	Fibroglandular	Fat	Water
Density (kg/m^3^)	1090	1032	985	998.2
Sound speed (m/s)	1615	1547	1465	1482
Characteristic impedance (× 10^6^ Pa.s/m)	1.760	1.597	1.443	1.479
Attenuation coefficient (dB/m/MHz)	–	60	40	0.22

**Fig. 1:**
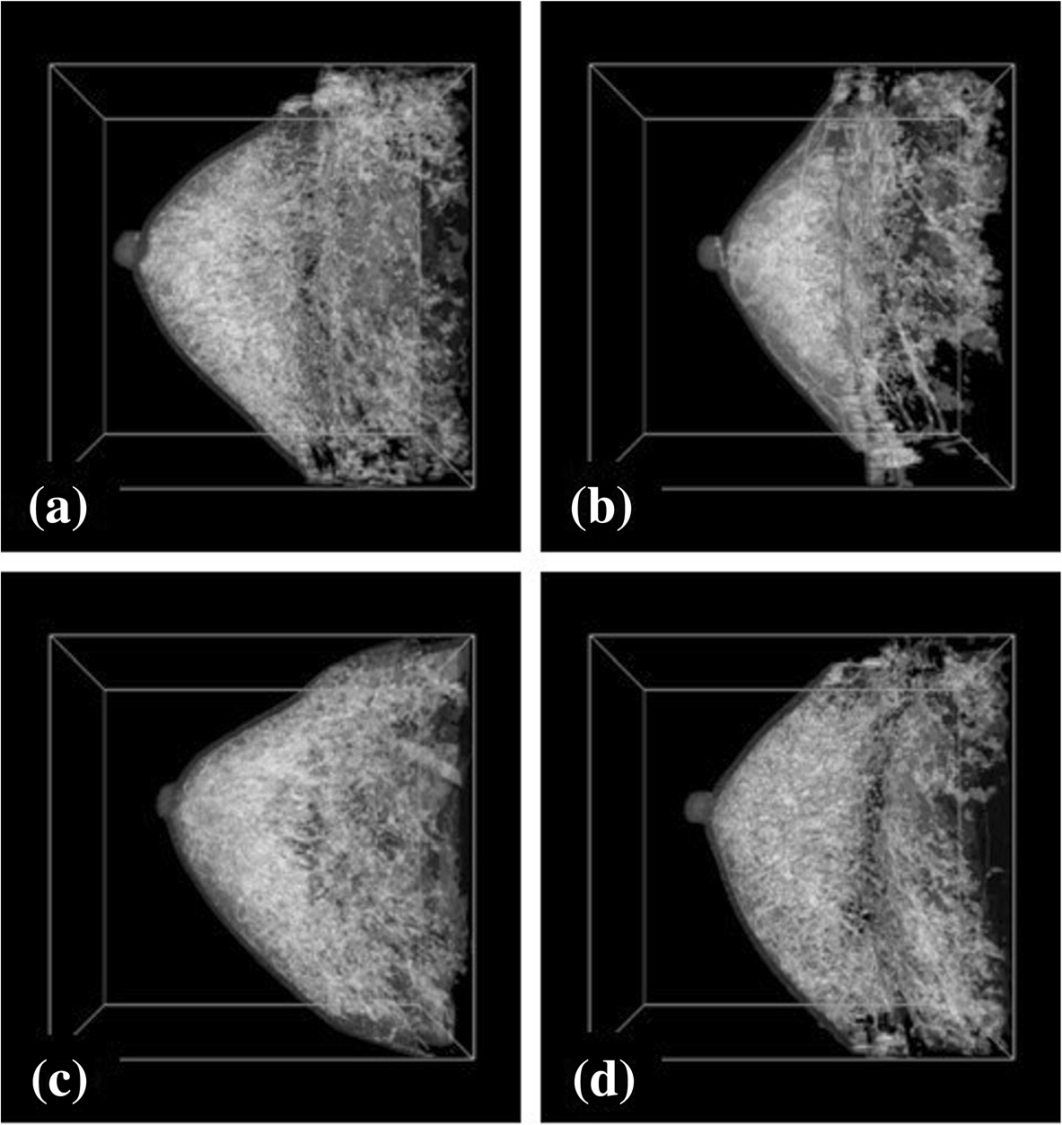
(**a**)-(**d**) Four samples of the breast phantoms based on the MRI data obtained from 12 patients

Breast phantoms were constructed from the MRI data of 12 patients using the described method, and were used as the input data for the breast HIFU treatment simulation to examine the effects of breast acoustic inhomogeneity on the focal error. Figure 1 shows four samples of breast phantoms which were represented by the iso-surface of the threshold brightness with opacity of 20%. MRI data were obtained as T1-weighted contrast-enhanced MR images by 1.5 T MRI scanner (GE Health Care), and were resolved at 0.625 × 0.625 mm and sliced to 0.8 mm. Breast fat percentage was assumed to be 66.5% [18] for every breast phantom to eliminate the effects of the arbitrary manipulations during the segmentation processes, although this rate varies among patients. The mean age of all patients was 47.3 ± 6.7 years (mean ± SD, range 39 to 57 years). The mean threshold brightness value used for the segmentation of fat and fibroglandular tissue varied from 194 to 438 for the created breast phantoms, and the mean threshold brightness was 255.1 ± 63.4 (mean ± SD). The correlation coefficient between the age of the patients and the threshold brightness was − 0.52.

### HIFU simulation

A breast HIFU simulation was executed using the HIFU simulator ZZ-HIFU developed as a simulation code for the propagation of the ultrasound wave emitted from a HIFU device through tissues showing varying acoustic properties [19–21]. Among the basic equations, the momentum equation for multi-media with constitutive equation for viscous fluid or viscoelastic body was solved to reproduce the propagation of the ultrasound wave through inhomogeneous media considering their nonlinearity. Constitutive equation for viscous fluid was used because transversal waves through soft tissues have a speed much lower than the speed of sound. Heat equation with the viscous dissipation as a heat source was also solved to obtain temperature distribution.

Basic equations are discretized using finite difference method by the 6th order central difference scheme in space and were temporally developed based on the FDTD method. The code was parallelized for large-scale computing by thread parallelization using OpenMP and process parallelization using MPI with domain decomposition. A tune-up code for K computer (Riken Advanced Institute for Computational Sciences; RIKEN AICS, Japan) called ZZ-HIFU-K showing an effective performance over 20% by 24,576 nodes was achieved. In the present study, 1024 nodes of the K computer were employed for the breast cancer HIFU treatment simulations.

In our previous study [15], it was validated that the focused ultrasound waves propagating through inhomogeneous media with known shape and physical properties can be simulated by ZZ-HIFU in quantitative agreement with the experimental results. Therefore, the accuracy of ultrasound propagation simulation by HIFU depends on both the accuracy of the digital phantom constructed using image-based modeling and the physical properties of tissues.

### Numerical model

**Fig. 2:**
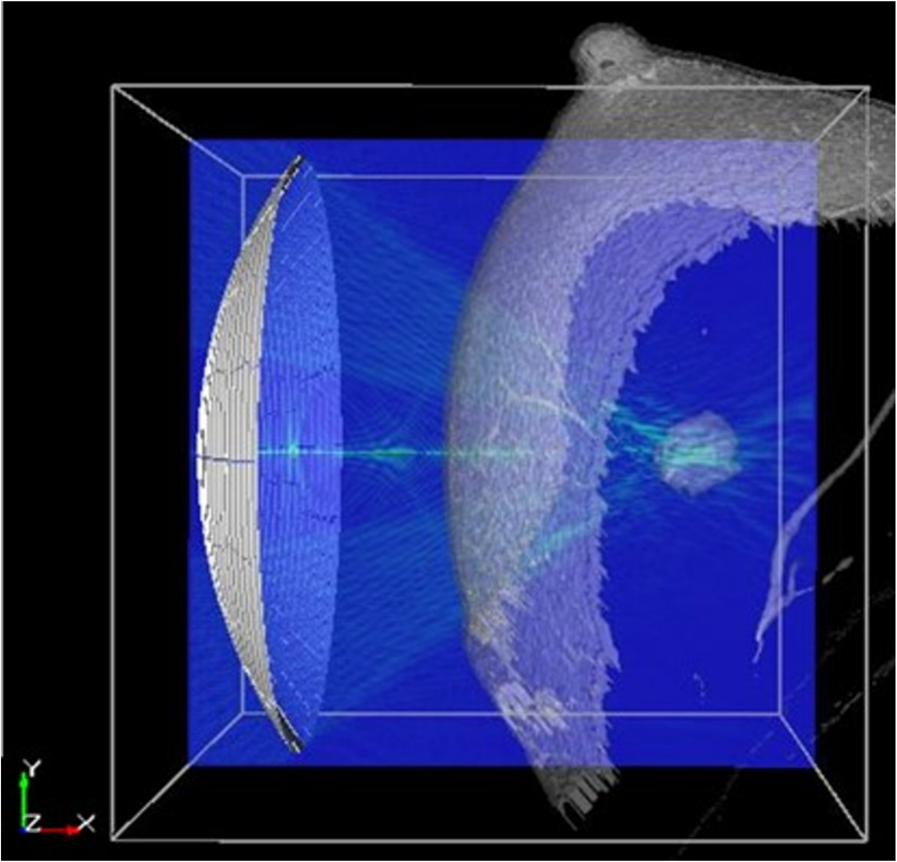
Numerical domain for the breast cancer HIFU treatment simulation. Geometrical focus of a 256ch phased-array transducer is assigned to the target in a breast phantom

Figure 2 shows an arrangement of a 256-ch phased-array transducer to a breast phantom with an ultrasound field on a cross-section. The transducer was designed for the f-number of 5/6 with a center hole for ultrasound imaging probe. The focal distance and diameter of the transducer were 100 mm and 120 mm, respectively, and the diameter of the center hole was 35 mm. The frequency was 2 MHz. The numerical domain size was 128 × 128 × 128 mm, which was resolved by 2560 × 2560 × 2560 points with the orthogonal mesh with the grid size of 0.05 mm. The wave length of 2 MHz ultrasound in water is 0.75 mm, which was resolved with 15 grid points. The numerical domain was decomposed into 16 × 8 × 8 for parallel computing.

Because the resolution of the breast phantom constructed using the MRI data was shown to be lower than the resolution of the HIFU simulation, the input data of the body for the breast HIFU treatment simulation was generated through the oversampling using tri-linear interpolation for brightness values or the nearest neighbor interpolation for index labels.

### Localized heating ratio and focusing ratio

**Fig. 3:**
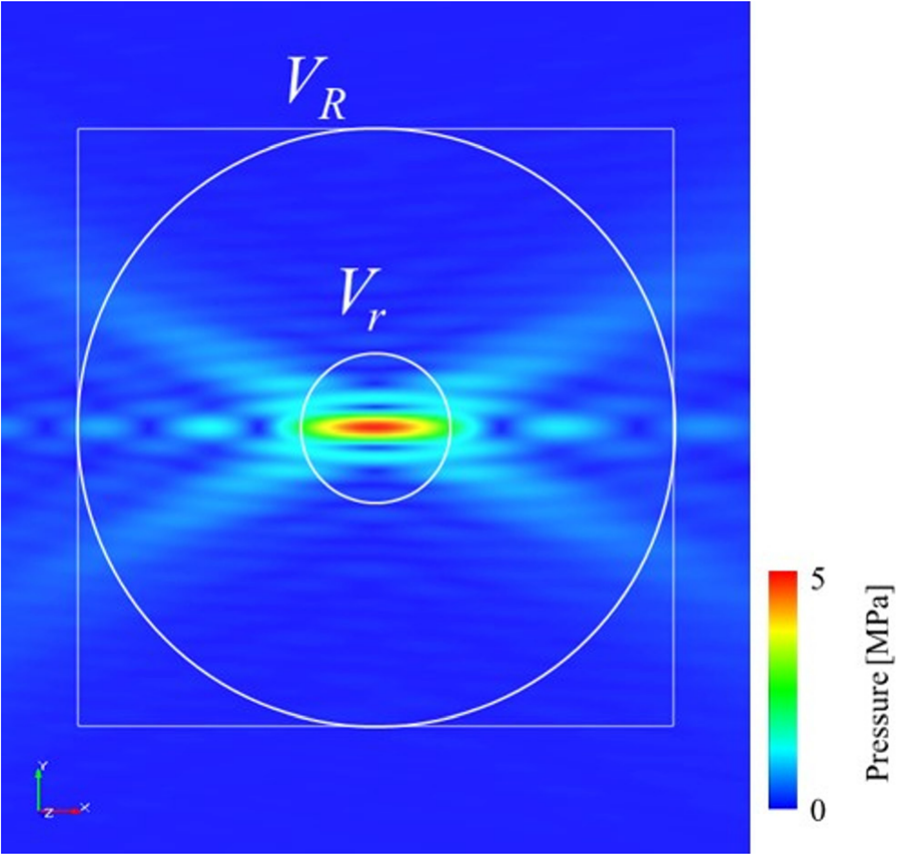
Ultrasound field around focus in the case of the HIFU simulation without breast phantom. Circles indicate the spherical volume regions V_R_ with radius of 10 mm and V_r_ with radius of 2.5 mm respectively. Square line indicates a region of interest for discussing on focal shape

In Fig. 3, the ultrasound field on the cross-section along the symmetry axis of the HIFU beam obtained from the HIFU simulation without breast tissue. Here, to evaluate how much the heat *Q* is concentrated on the target, a localized heating ratio *β* was defined as

1 $$ \beta =\frac{\int_{V_r} QdV}{\int_{V_R} QdV} $$

where the volumes of the localized spherical region are *V*_*r*_ and that of the entire spherical region *V*_R_ (Fig. 3). Because the heat due to viscous dissipation corresponding to the attenuation is taken into account in this study, the localized heating ratios were calculated directly using Eq. (1). In addition, *R* = 10 mm and *r* = 2.5 mm were selected for the evaluation of the localized heating ratio, although the value of the localized heating ratio depends on the volumes *V*_*r*_ and *V*_R_. If the heat distributes uniformly, the ratio becomes *β* = 0.0156. In the case presented in Fig. 3, the localized heating ratio without breast tissue object becomes *β* = 0.232. Therefore, the increased value of the localized heating ratio indicates that more heat is generated around the focal region at the target. The heat Q can be represented as:

2 $$ Q=\alpha \frac{p^2}{\rho c} $$

where *α* is the attenuation coefficient, p pressure, *ρ* density, and c sound speed. Eq. (2) indicates that the localized heating ratio depends on the distribution of the ultrasound wave and the distribution of the attenuation coefficient. The localized heat ratio can represent a useful indicator for the treatment efficacy, but it is impossible to distinguish the cases between the high attenuation with low ultrasound intensity and the low attenuation with high ultrasound intensity. To evaluate the focal error, a focusing ratio *ϕ* can also be defined as:

3 $$ \phi =\frac{\int_{V_r}\frac{p^2}{\rho c} dV}{\int_{V_R}\frac{p^2}{\rho c} dV} $$

The focusing ratio *ϕ*_*0*_ in the case without breast tissue object (Fig. 3) becomes *ϕ*_*0*_ = 0.243. A normalized focusing ratio *Φ*, using the value of the focusing ratio *ϕ*_*0*_, can be defined as *Φ = ϕ*/*ϕ*_*0*_.

## Results

**Fig. 4:**
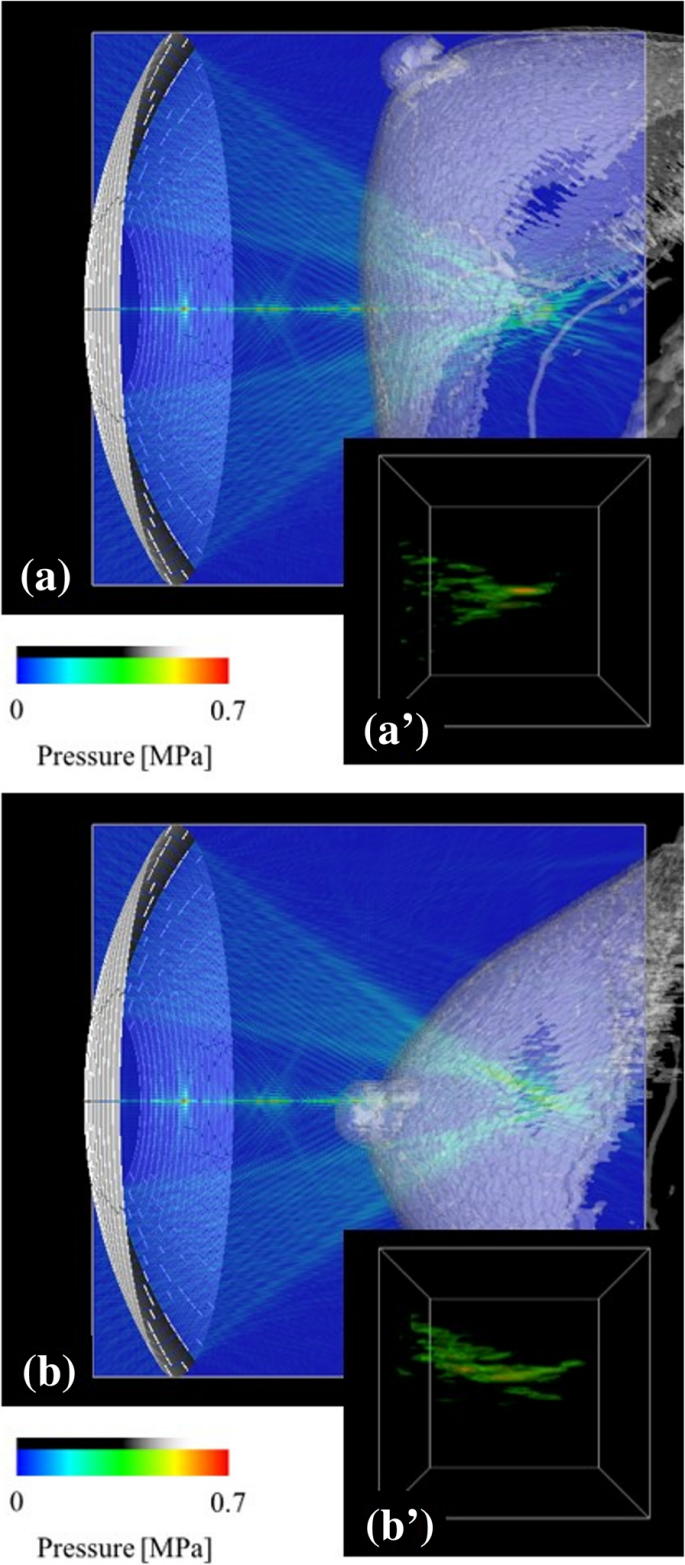
Ultrasound field and focal shape in the HIFU simulation for different transducer arrangements of (**a**) case A (*Φ* = 0.384) and (**b**) case B (*Φ* = 0.386), using the breast phantom of Fig. 1(a)

The results of the breast tissue HIFU treatment simulations for case A and B are presented in Fig. 4(a) and (b), respectively. The differences of the cases were the target position and the transducer arrangement, the cases were not for the conditions of the actual treatment but virtual ones. The ultrasound field was visualized in a cross-section. The ultrasound wave was shown to propagate from the phased-array transducer through the skin to the target in the breast tissue (Fig. 4(a) and (b)). The focal shape is presented as a high-pressure amplitude region around the focus, which was visualized using the volume rendering method (Fig. 4(a’) and (b’)). The size of the volume rendering region is 20 × 20 × 20 mm. The focal shape was distorted due to the reflection and refraction of the ultrasound wave at the tissue interface. As shown in Fig. 4(b), the arrangement of the array transducer with the nipple, placed on the acoustic axis, leads to a higher distortion of the focal shape (Fig. 4(b’)). The focusing ratios for the case A (Fig. 4(a)) and case B (Fig. 4(b)) were *ϕ* = 0.093 and *ϕ* = 0.094, respectively. Both values are considerably lower than those obtained for the case without the breast model (*ϕ*_*0*_ = 0.243). Therefore, the normalized focusing ratios for the case A and case B were shown to be *Φ* = 0.384 and *Φ* = 0.386, respectively.

**Fig. 5:**
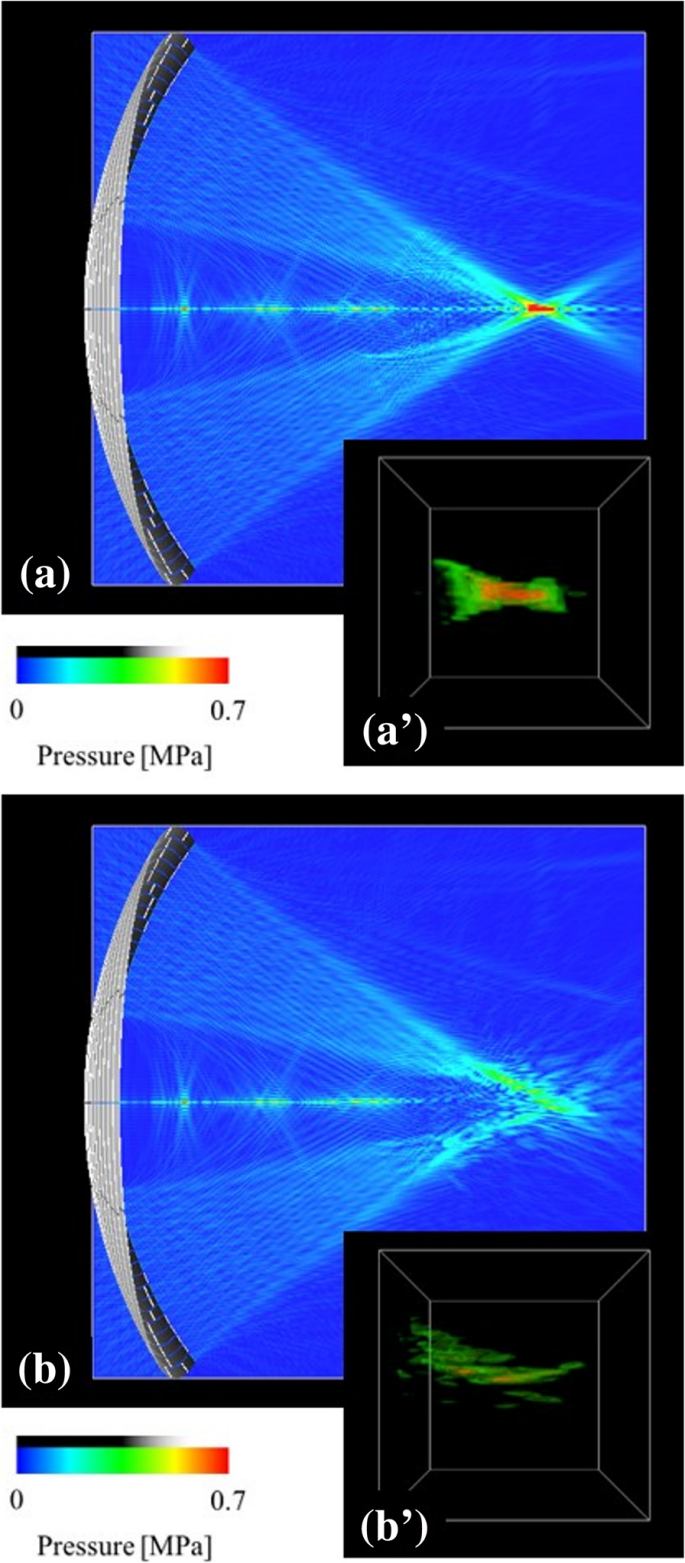
Ultrasound field and focal shape in the HIFU simulations for the case B of Fig. 4, using the breast phantom without (**a**) fibroglandular tissue or (**b**) skin

With keeping the breast model and the arrangement of the HIFU transducer the same as those for the case B (Fig. 4(b)), we considered two different breast models to examine the tissue effects on the focal distortion, i.e., the breast model without fibroglandular tissue, where the physical properties of fat were applied for fibroglandular tissue, and the model without skin, using the physical properties of water as those of the skin. The ultrasound field and focal shape without the fibroglandular tissue are presented in Fig. 5(a) and (a’), and although the characteristic impedance of the skin was shown to be considerably higher than that listed in Table 1, this did not affect the focusing. In contrast, when using the model without skin, the focal shape was shown to be highly distorted (Fig. 5(b) and (b’)) similar to that presented in Fig. 4(b’).

We applied the focus control based on the time-reversal method for the breast cancer HIFU treatment simulation. Initially, the emitted ultrasound propagated through the tissue. Following this, control parameters, the phase shift and amplitude, for each element of the phased-array transducer were obtained using the cross-correlation of the ultrasound recorded by each element of the array transducers and a reference one. Afterward, ultrasound was emitted from the phased-array transducers using the control parameters.

**Fig. 6:**
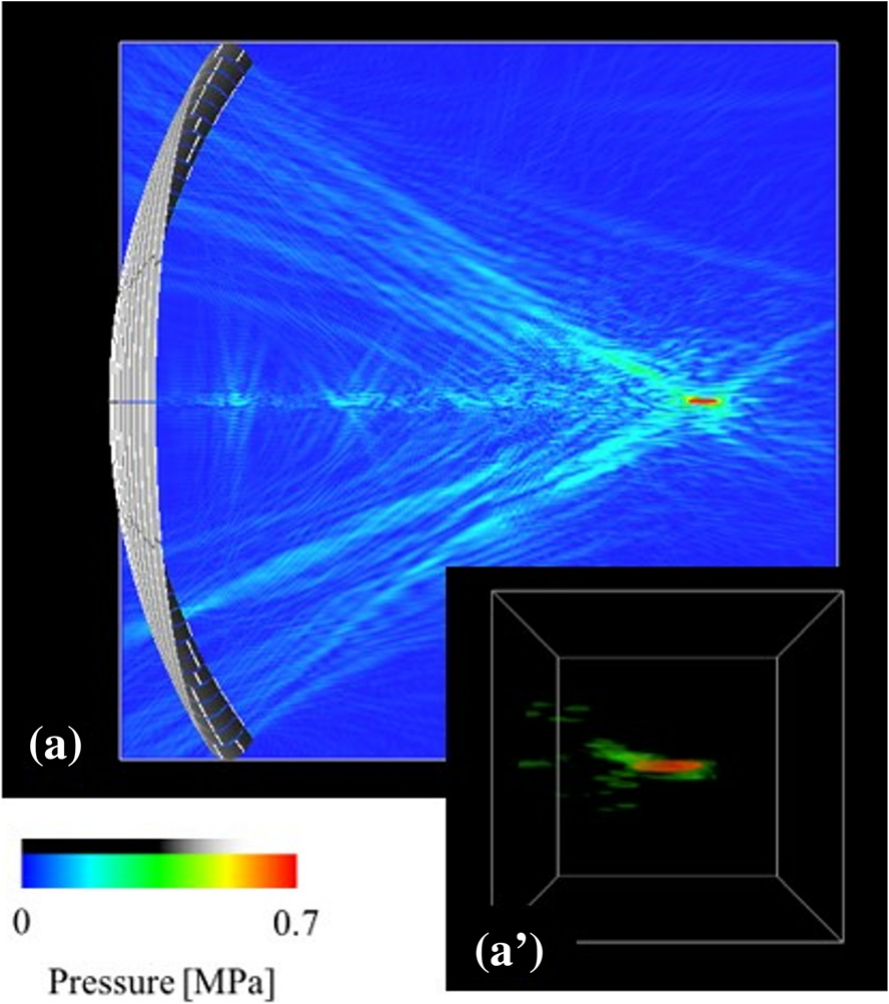
Focus quality obtained by the focus control based on the time-reversal in the HIFU simulation for the case B of Fig. 4 (*Φ* = 0.543) ((**a**) ultrasound field, (**a'**) focal shape)

In Fig. 6, the ultrasound field with focus control is presented, showing that the ultrasound wave is focused on the target correctly. The focal shape, particularly the high-pressure amplitude (Fig. 6(a’), red) was shown to be considerably clearer than that observed in the case presented in Fig. 4(b’). The normalized focusing ratio improved to *Φ* = 0.543, which is considerably higher than that previously obtained, *Φ* = 0.386 (Fig. 4(b)).

**Fig. 7:**
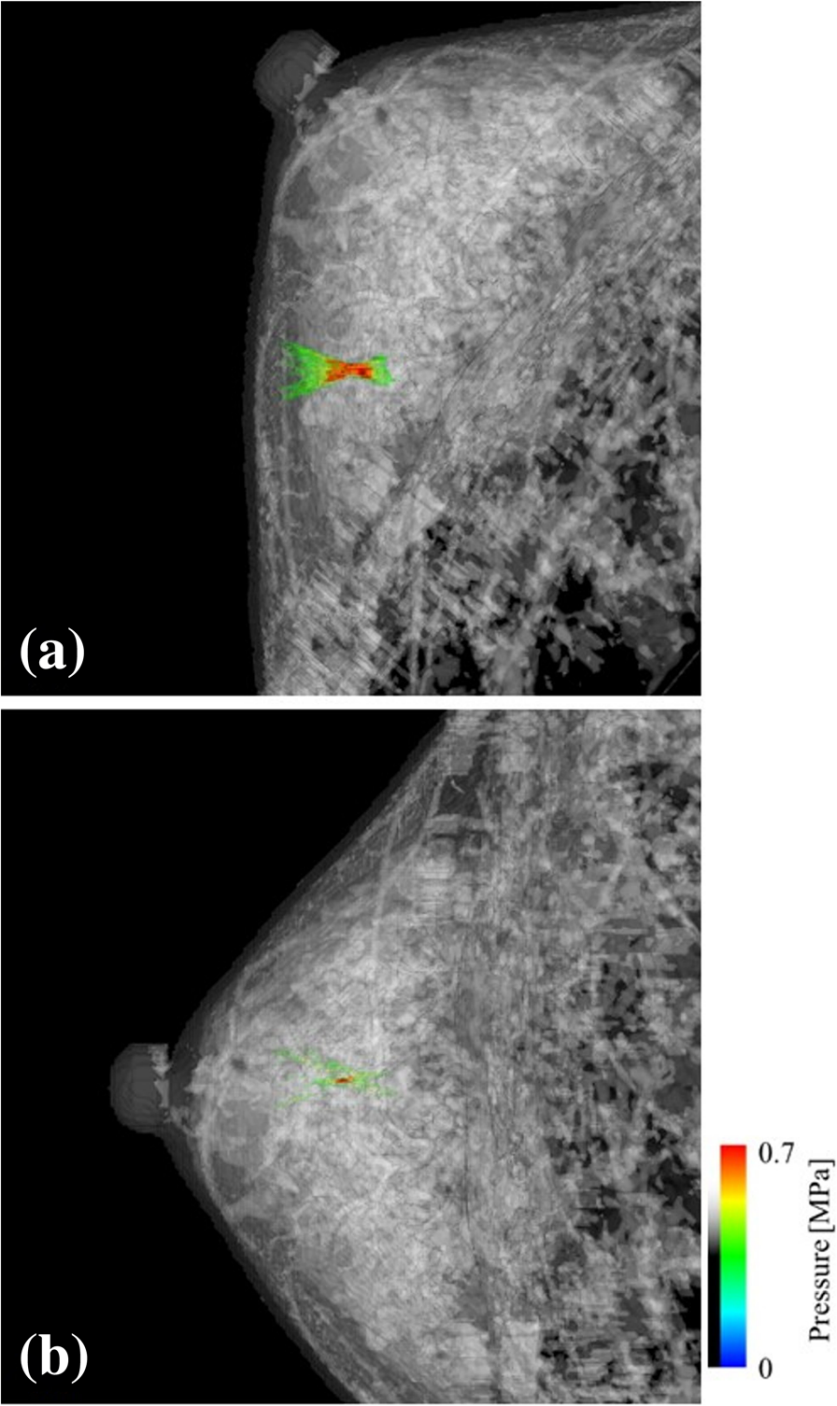
Comparison of the focal shape depending on the target position and the transducer arrangement in the HIFU simulation, using the breast phantom of Fig. 1(b) ((**a**) *Φ* = 0.901, (**b**) *Φ* = 0.465)

We compared the relationship between the fibroglandular structures and focal shapes between two possible target arrangements (Fig. 7). The focal shape presented in Fig. 7(a) is much clear than that presented in Fig. 7(b). In the former, no fibroglandular tissue can be observed between the skin and focus (Fig. 7(a)), whereas in the latter, the focus is deep in the fibroglandular tissue and highly distorted (Fig. 7(b)). The normalized focusing rate for both cases were shown to be *Φ* = 0.901 and *Φ* = 0.465, respectively.

**Fig. 8:**
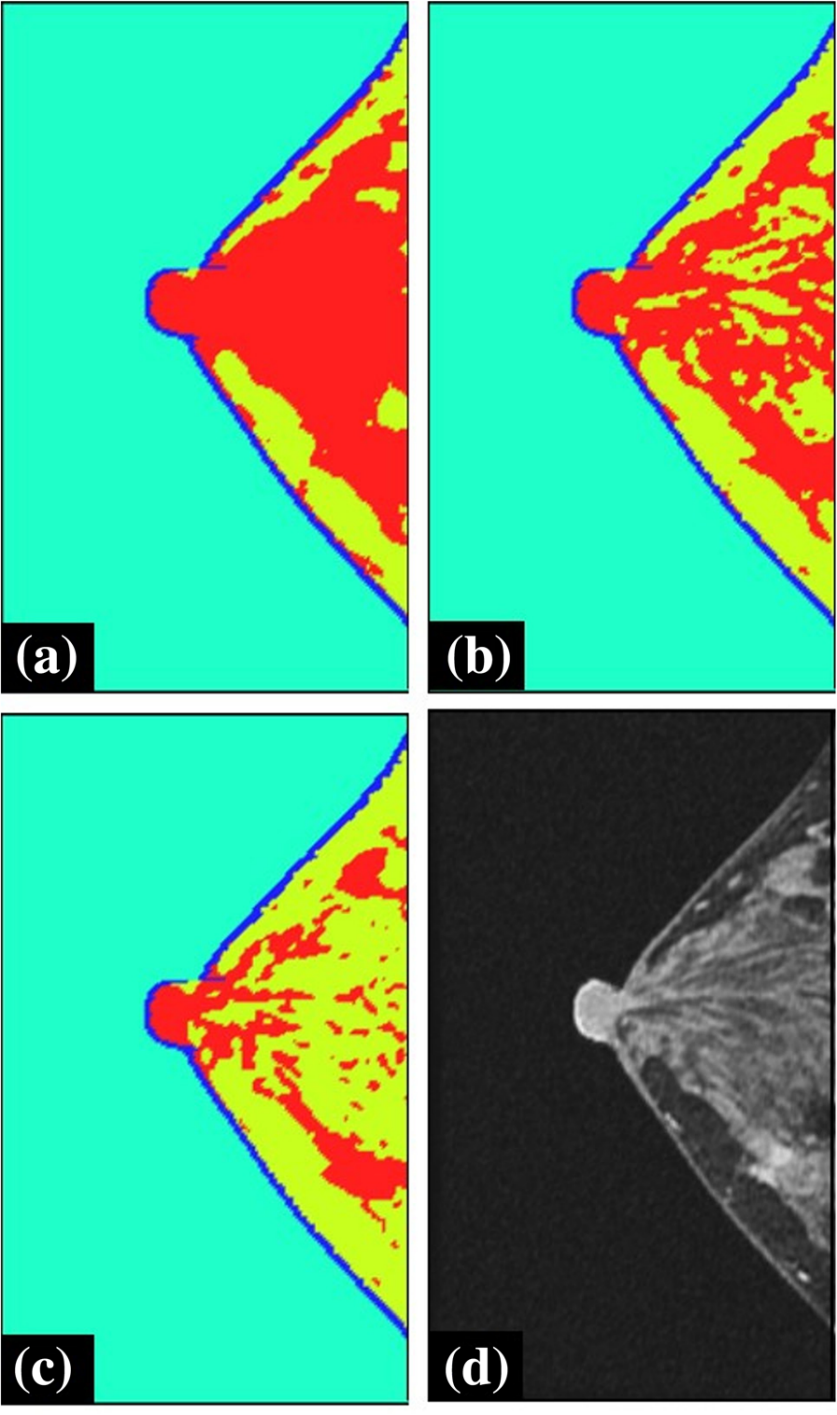
Dependency of the obtained breast structure on the brightness threshold value selected for the segmentation of the fibroglandular tissue (red) and fat (yellow), presented using breast phantom of Fig. 1(b). Brightness threshold values of (**a**) 301, (**b**) 437, and (**c**) 528 correspond to the fat percentage of 46.5, 66.5, and 86.5%, respectively. (**d**) Original MR image

The brightness threshold for the segmentation of fat and fibroglandular tissue was calculated based on the fat ratio in breasts, and we investigated the relationship between the selected brightness thresholds on the breast model obtained. We compared breast structures obtained using the brightness thresholds of 301, 437, and 528, which corresponded to the fat rates 46.5, 66.5, and 86.5%, respectively (Fig. 8(a), (b) and (c)). Figure 8(d) was original MR image. The brightness threshold of the breast phantom was higher than that of the other breast phantoms.

**Fig. 9:**
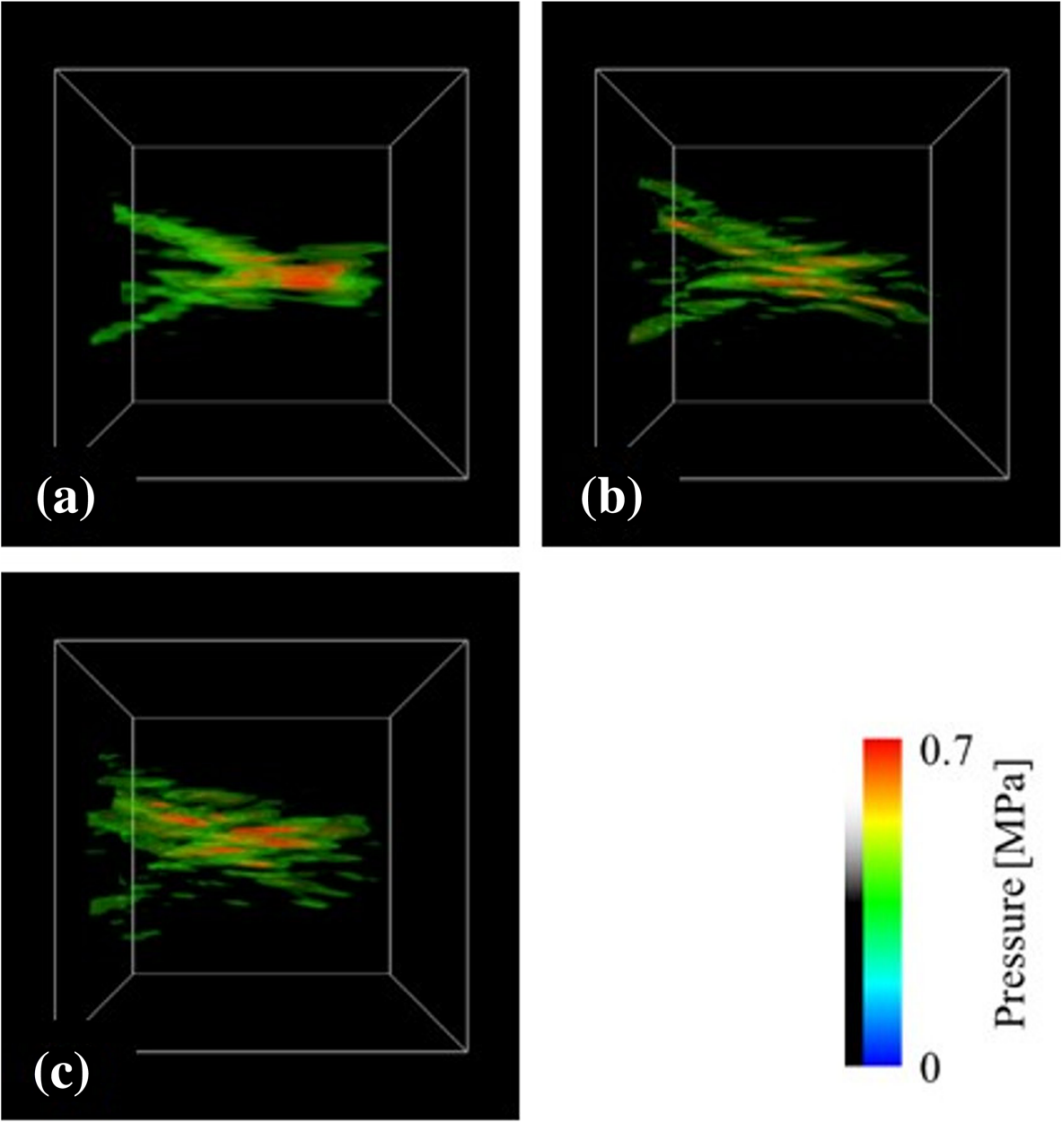
Dependency of the focal shape on the brightness threshold value selected for the segmentation of the fibroglandular tissue and fat. Brightness threshold values of (**a**) 301, (**b**) 437, and (**c**) 528 correspond to the breast models of Fig. 8(a), (b) and (c), respectively ((a) *Φ* = 0.704, (b) *Φ* = 0.465, (c) *Φ* = 0.478)

In Fig. 9, we presented the focal shape comparisons among the breast phantoms obtained using different brightness thresholds, as shown in Fig. 8, with keeping the breast model and the arrangement of the HIFU transducer the same as those for the case presented in Fig. 7(b). The normalized focusing ratios obtained here were *Φ* = 0.704, 0.465, and 0.478 (Fig. 9(a), (b), and (c), respectively).

**Fig. 10:**
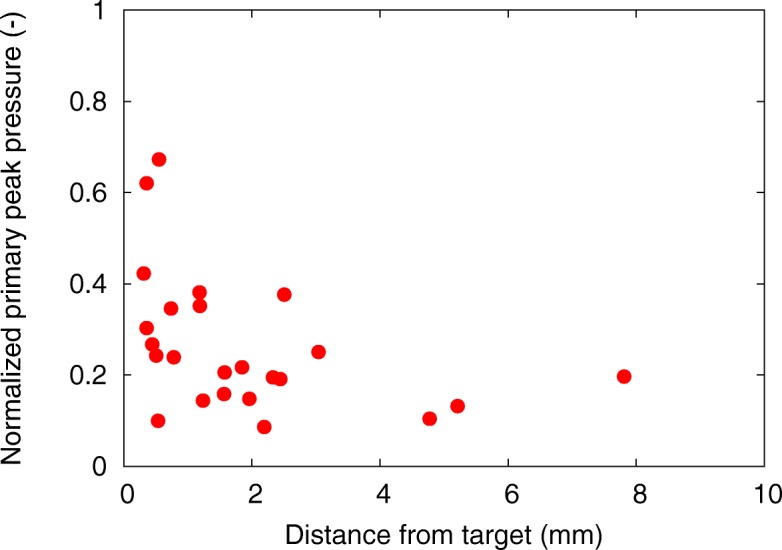
Normalized primary peak pressure as a function of the distance from the target to the peak position for 24 breast HIFU treatment simulations

Figure 10 shows the relationship between the normalized primary peak pressure and the distance from the target to the position of the primary peak pressure. The results obtained using 24 breast HIFU treatment simulations for 12 breast models and two target positions were plotted, and the primary peak pressure was normalized to that obtained using the HIFU simulation without breast tissue object. As the primary peak pressure decreased due to the attenuation and scattering during wave propagation through the breast, the distance from target was shown to spread to 8 mm. As shown in Figs. 4(b’), 9(b) and (c), pressure peaked locally at various points. The primary peak can be recorded far from the target by decreasing the primary peak pressure. The largest distance from a target was 7.8 mm, which was observed in a simulation using the breast model of Fig. 1(c). However, other simulations using the same model had the primary peak at 1.6 mm from the target.

**Fig. 11:**
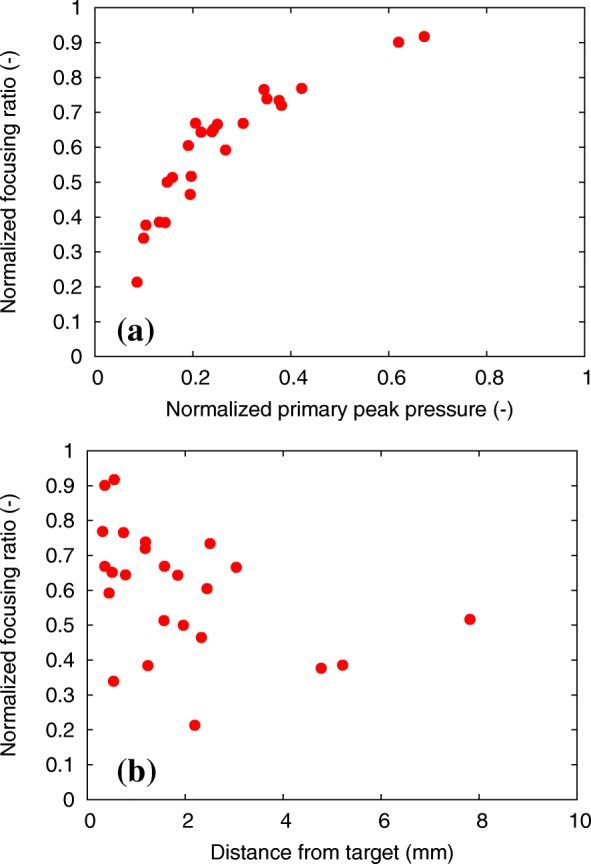
Normalized focusing ratio as the function of (**a**) the normalized primary peak pressure and (**b**) distance from target to the peak position for 24 breast HIFU treatment simulations

The correlation coefficient between the localized heating ratio and the focusing ratio is 0.99 in the present study, because the attenuation coefficient of fibroglandular tissue was similar to that of fat tissue (Table 1). Here, the dependency of the focusing ratios for the primary peak pressure and the distance from the target to the position of the primary peak pressure are plotted in Fig. 11. The focusing ratio was shown to be highly correlated with the normalized primary peak pressure because the high focusing ratio results in the increase in the primary peak pressure. In addition, for the normalized focusing ratio higher than *Φ* = 0.6, it distributes within 3-mm distance. However, the lower normalized focusing ratios do not depend on the distance from the target, because the pressure can take local peaks at various points.

**Fig. 12:**
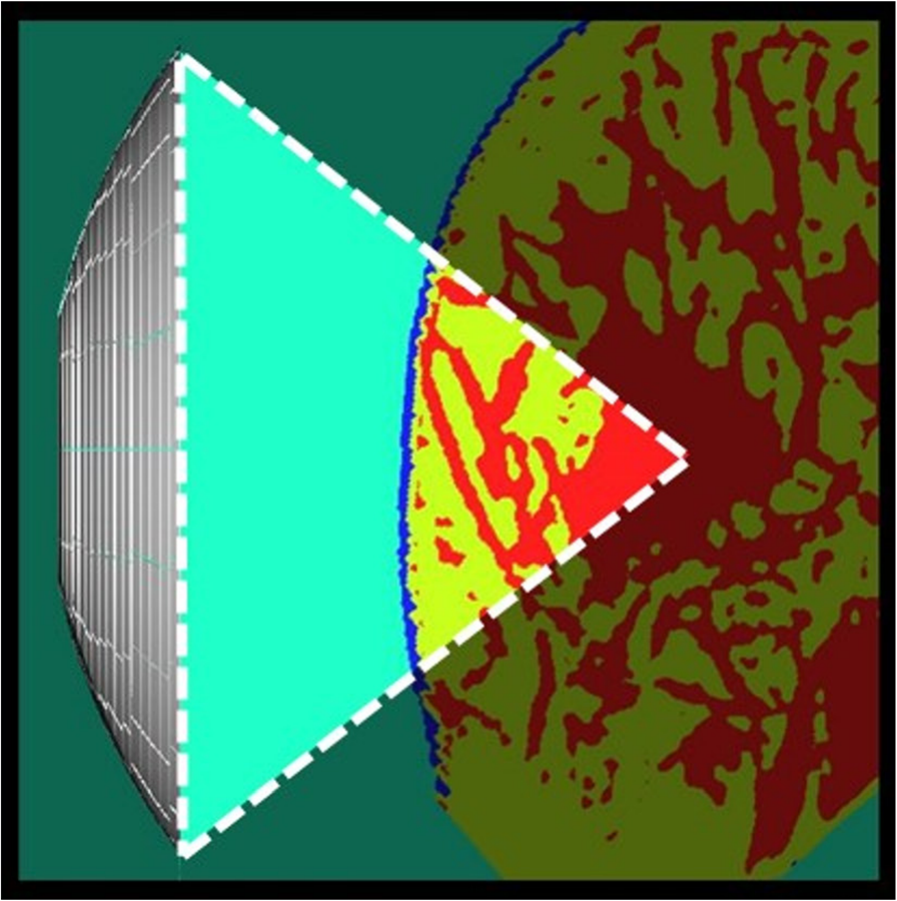
Region for calculating local acoustic inhomogeneity (indicated by the white dashed line)

Here, we proposed an indicator to determine the effects of the breast structure on the HIFU focal error. One section of the breast tissue on the ultrasound wave path from the transducer to the target was analyzed (Fig. 12). Considering the acoustical inhomogeneity related to the gradient of characteristic impedance on the ultrasound path, local acoustic inhomogeneity χ was defined for the extracted region *V* on the ultrasound path as

4 $$ \chi =\frac{\sqrt{\frac{1}{V}{\int}_{\Omega}{\left|\nabla \left(\rho c\right)\right|}^2 dV}}{\rho_w{c}_w/{\lambda}_w} $$

where, *ρ*_w_, *c*_w,_ and *λ*_w_ are water density, sound speed, and wavelength in water, respectively.

**Fig. 13:**
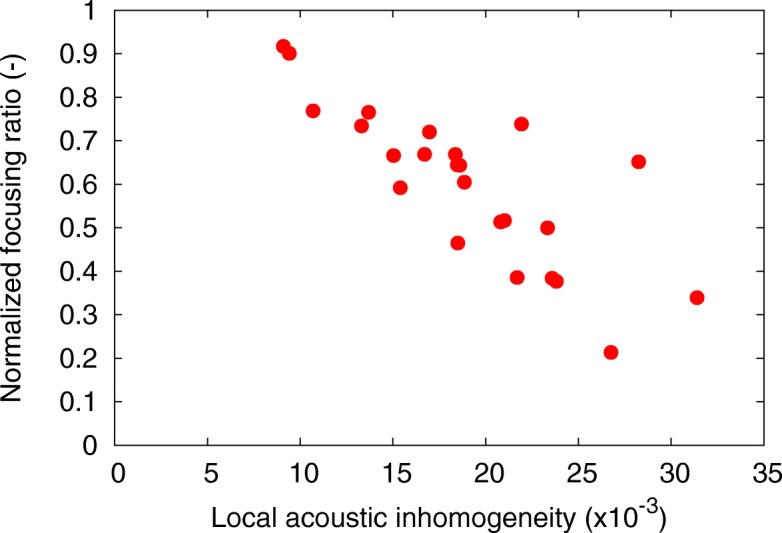
Normalized focusing ratio as the function of local acoustic inhomogeneity, obtained for 24 breast HIFU treatment simulations. Correlation coefficient is − 0.80

**Fig. 14:**
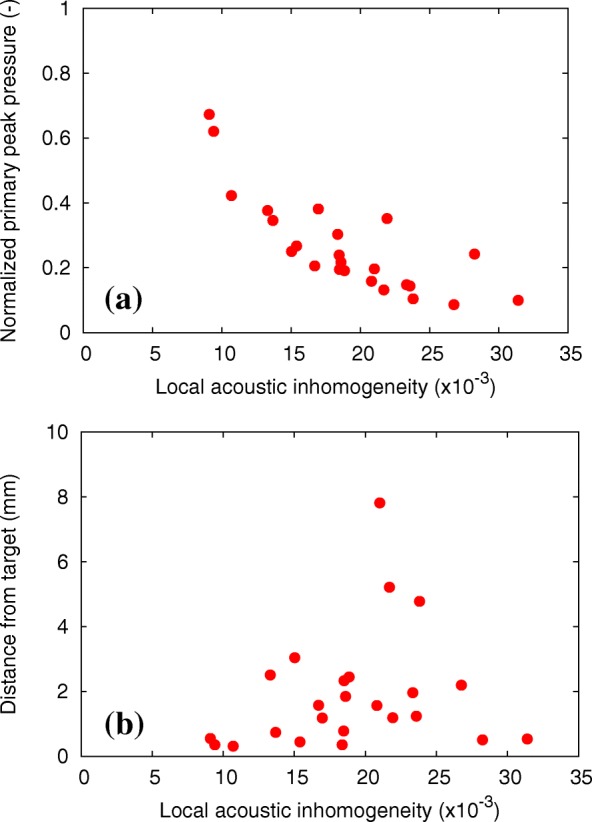
(**a**) Normalized primary peak pressure and (**b**) distance from the target to the peak position as the function of local acoustic inhomogeneity for 24 breast HIFU simulations

The relationship between the normalized focusing ratio and the local acoustic inhomogeneity is shown in Fig. 13. Figure 14 shows the normalized primary peak pressure and the distance from the target to the primary peak pressure as the function of the local acoustic inhomogeneity.

## Discussion

We demonstrated that the focal shapes are distorted depending on the target position and the arrangement of the transducer. Numerical simulation results showed that the complex distribution of fibroglandular tissue leads to the distortion of focus. As shown in Figs. 4, 5 and 7, the structure of the fibroglandular tissue affects ultrasound focusing, and an arrangement of the HIFU transducer such that the ultrasound wave avoids the fibroglandular tissue is necessary.

Therefore, if the cancer is located deep in the fibroglandular tissue, the focus control using the phased-array transducer is required for the safe and efficient treatment of breast cancer. As shown in Fig. 6, numerical simulations demonstrated that the clear focus and high focusing ratios can be obtained using the focus control based on the time-reversal method with the phased-array transducer, allowing a safe and efficient HIFU treatment of breast cancer. In general, without numerical simulations, it is not easy to obtain non-invasively the control parameters of the phased-array transducer for beam steering in inhomogeneous media because the time-reversal approach requires a sound source at a target position.

In Fig. 8, the network structure of the fibroglandular tissue was reproduced by using the selected brightness threshold. The segmented breast model seems to be in good agreement with the original MR image (Fig. 8(d)). With the decrease in the brightness thresholds, the small fibroglandular structures disappears. In Fig. 9, with the increase in the brightness threshold, the normalized focusing ratio decreases and the focal distortion increases. The relationship between the fibroglandular small structure scales and the ultrasound wavelength was shown to be important for the ultrasound propagation, although these differences in our model depended on the artificially selected brightness threshold. The simulation results included errors due to the breast modeling, because the breast structure depends on the personal as high or low fatty. However, the distance from the target to the peak position was shown to depend on the target position and the arrangement of the transducer rather than the breast phantom used as shown in Fig. 7. Thus, it is considered that the focal errors vary as shown in Fig. 10, which reduces localized heating because of the decrease of the focusing ratio (Fig. 11(b)).

The arrangement of the transducer to a target is arbitral for the freedom of rotation even if the geometrical focus of the transducer is fixed on the target. Thus, focal shapes are distorted depending on the target position as well as the arrangement of the transducer despite breast tissue representing soft tissue. The numerical simulation results state that the complex distribution of fibroglandular tissue causes the distortion of focus. In addition, during the automatic HIFU treatment, the device must determine the optimal arrangement of the transducer considering its safety and efficacy. Therefore, the identification of the optimal arrangement that leads to the reduction of the HIFU focal error depending on the breast structure is desired before the initiation of the treatment.

In Fig. 13, the increase in the normalized focusing ratio was shown to highly correlate with a decrease in the local acoustic inhomogeneity (correlation coefficient, − 0.80). In Fig. 14, the normalized primary peak pressure increases with a decrease in the local acoustic inhomogeneity (correlation coefficient, − 0.80). In contrast, the distance from the target to the primary peak pressure was shown to be independent of the local acoustic inhomogeneity. However, when analyzing samples with the local acoustic inhomogeneity lower than χ = 15 × 10^− 3^, the primary peak pressure was shown to distribute within 4 mm from the target. Therefore, both a high focusing rate and a low focal error can be obtained if the local acoustic inhomogeneity is low. This suggests that the optimal arrangement of the transducer to the target during the breast cancer HIFU treatments can be achieved by minimizing the local acoustic inhomogeneity.

## Conclusions

Breast HIFU treatments were simulated using 12 digital breast models constructed from the MRI data obtained from 12 patients, showing that the focal shapes are distorted even when the waves propagated through soft tissue, such as breast tissue. Focal error was shown to be caused by the complex distribution of fibroglandular tissue and to depend on the target position as well as the arrangement of the transducer, although focal error also depends on several transducer parameters such as frequency and f-ratio. On the other hand, to obtain the optimal arrangement of the transducer to the target before the initiation of the breast cancer HIFU treatment, the local acoustic inhomogeneity may be used as an indicator value to reduce the HIFU focal error. We showed that the focusing ratio corresponding to the contrast heating ratio increases with a decrease in the local acoustic inhomogeneity. This suggests that the optimal arrangement of the transducer to the target for the breast cancer HIFU treatments is achieved by minimizing the local acoustic inhomogeneity. Because the modeling of the digital phantom of breast includes uncertainties about tissue segmentation and physical properties, further improvements in the modeling are required to obtain more accurate HIFU treatment simulations. In addition, since the acoustic properties of tissues highly depend on the temperature, the full simulation for the breast HIFU treatment requires the coupling of the acoustic simulation and the thermal simulation at the treatment time scale.

## Abbreviations

FDTD: Finite difference time domain
HIFU: High-intensity focused ultrasound
MRI: Magnetic resonance imaging
